# Electrical, structural, and optical properties of sulfurized Sn-doped In_2_O_3_ nanowires

**DOI:** 10.1186/s11671-015-0995-z

**Published:** 2015-08-01

**Authors:** M. Zervos, C. N. Mihailescu, J. Giapintzakis, A. Othonos, A. Travlos, C. R. Luculescu

**Affiliations:** Nanostructured Materials and Devices Laboratory, Department of Mechanical and Manufacturing Engineering, P.O. Box 20537, Nicosia, 1678 Cyprus; Nanotechnology Research Center (NRC), P.O. Box 20537, Nicosia, 1678 Cyprus; Laboratory of Ultrafast Science, Department of Physics, University of Cyprus, P.O. Box 20537, Nicosia, 1678 Cyprus; NCSR Demokritos, Institute of Nanoscience and Nanotechnology, 153 10 Aghia Paraskevi, Athens, Greece; National Institute for Laser, Plasma and Radiation Physics, Str.Atomistilor, P.O. Box MG-36, 077125 Magurele, Romania

**Keywords:** Sulfur, Indium tin oxide, Nanowires

## Abstract

Sn-doped In_2_O_3_ nanowires have been grown on Si via the vapor-liquid-solid mechanism at 800 °C and then exposed to H_2_S between 300 to 600 °C. We observe the existence of cubic bixbyite In_2_O_3_ and hexagonal SnS_2_ after processing the Sn:In_2_O_3_ nanowires to H_2_S at 300 °C but also cubic bixbyite In_2_O_3_, which remains dominant, and the emergence of rhombohedral In_2_(SO_4_)_3_ at 400 °C. The resultant nanowires maintain their metallic-like conductivity, and exhibit photoluminescence at 3.4 eV corresponding to band edge emission from In_2_O_3_. In contrast, Sn:In_2_O_3_ nanowires grown on glass at 500 °C can be treated under H_2_S only below 200 °C which is important for the fabrication of Cu_2_S/Sn:In_2_O_3_ core-shell p-n junctions on low-cost transparent substrates such as glass suitable for quantum dot-sensitized solar cells.

## Background

Semiconductor nanowires (NWs) are attractive for the fabrication of nanoscale devices such as nanowire solar cells (NWSCs) and sensors, but some of the main issues pertaining to the realization of high performance devices is doping and control or modification of the surface properties which are important in view of the large surface to volume ratio [1]. For instance the surface modification of III–V NWs such as InAs and GaAs NWs using sulfur has been shown to improve their electrical and optical properties [2–6], but the effect of sulfur on the structural, electrical and optical properties of metal oxide (MO) NWs has not been considered previously, despite the fact that it leads to a suppression of surface recombination and improvement of the photoluminescence (PL) in bulk ZnO [7, 8]. Controlling the surface properties of MO NWs is also necessary in order to suppress the adsorption and desorption of oxygen which is responsible for charge fluctuations and has been achieved so far by using polyimide and polymethyl methacrylate on ZnO and SnO_2_ NWs, respectively [9, 10].

Recently, we carried out a systematic investigation into the growth and properties of Sn-doped In_2_O_3_ or indium tin oxide (ITO) NWs grown on Si by the vapor-liquid-solid (VLS) mechanism [11] which may be converted to metal oxysulfide (MOxS) NWs with different properties by post growth processing under H_2_S that is useful for NWSCs. It has been shown that bulk β-In_2_S_3-3x_O_3x_ has an optical band gap that varies from 2.1 eV in pure β-In_2_S_3_ to 2.9 eV when it contains 8.5 at.% of oxygen and has been proposed as an alternative to CdS buffer layers in CuIn_x_Ga_1-x_Se_2_ solar cells [12, 13]. In addition, the post growth processing of Sn-doped In_2_O_3_ NWs under H_2_S at different temperatures is important in understanding their properties and limitations as gas sensors which so far has been considered only up to 250 °C [14–16].

Hence, we carried out a systematic investigation into the structural, electrical, and optical properties of Sn-doped In_2_O_3_ NWs following post growth processing under H_2_S between 300 to 600 °C. We observe the existence of cubic bixbyite In_2_O_3_ and the formation of hexagonal SnS_2_ after processing the Sn-doped In_2_O_3_ NWs under H_2_S at 300 °C but also cubic bixbyite In_2_O_3_, which remains dominant, and the emergence of rhombohedral In_2_(SO_4_)_3_ at 400 °C. The Sn-doped In_2_O_3_ NWs maintain their metallic-like conductivities after exposure to H_2_S while we observed the emergence of PL at 3.4 eV corresponding to band edge emission from In_2_O_3_ in addition to the emission at 2.5 eV which is related to oxygen vacancies and states lying energetically in the upper half of the energy band gap of the as-grown Sn-doped In_2_O_3_ NWs. Besides the above, we have also grown Sn-doped In_2_O_3_ NWs on soda lime glass (SLG) at 500 °C, but we find that a significant deterioration in their conductivity occurs after exposure to H_2_S above 200 °C which might be related to Na ion diffusion as it was not observed in the case of the Sn-doped In_2_O_3_ NWs grown on Si. We discuss the importance of these findings for the fabrication of quantum dot-sensitized solar cells (QDSSCs) consisting of n-type MO NWs like SnO_2_, Sn:In_2_O_3_, and p-type chalcogenide semiconductors such as Cu_2_S or CuSnS_3_ [17].

## Methods

Sn-doped In_2_O_3_ NWs were grown on Si(001) and fused silica by low-pressure chemical vapor deposition (LPCVD) described in detail elsewhere [11]. Square samples of Si and fused silica ≈7 × 7 mm were cleaned sequentially in trichloroethylene, methanol, acetone, isopropanol, rinsed with de-ionized water, dried with nitrogen, and coated with ≈1 nm of Au. For the growth of Sn-doped In_2_O_3_ NWs, Sn (Aldrich, 2–14 Mesh, 99.9 %), and In (Aldrich, Mesh 99.9 %) were weighed with an accuracy of ± 1 mg. About 0.2 g of In containing ≈1 to 5 % wt Sn was used for the growth of the Sn-doped In_2_O_3_ NWs. Initially the LPCVD tube was pumped down to 10^−4^ mBar and purged with 600 sccm of Ar for 10 min at 1 mBar. Then the temperature was ramped up to 800 °C at 30 °C/min using the same flow of Ar. Upon reaching 800 °C, a flow of 10 sccm O_2_ was added in order to grow the Sn: In_2_O_3_ NWs over 60 min after which cool down took place over 30 min without O_2_. Note that the Sn-doped In_2_O_3_ NWs were grown on fused silica for 10 min in order to maintain transparency. The morphology of the Sn-doped In_2_O_3_ NWs was determined by scanning electron microscopy (SEM) while their crystal structure was determined by grazing incidence x-ray diffraction (GIXD) using a Rigaku Smart Lab diffractometer (9-kW rotating Cu-anode) with Cu-K_α1_ radiation. The Sn-doped In_2_O_3_ NWs were subsequently treated under a constant gas flow of 20 sccm Ar:50 sccm H_2_S between 300 to 600 °C for 60 min using a ramp rate of 10 °C/min but also at 400 °C for 30 min. All of the Sn-doped In_2_O_3_ NWs were inspected by SEM after post growth processing under H_2_S in order to determine changes in morphology while their crystal structure and phase purity was determined by GIXD. Transmission electron microscopy (TEM) was carried out using a FEI CM20 microscope operating at 200 kV while the constituent elements were identified by energy dispersive x-ray analysis (EDX) using a FEI SEM Inspect S equipped with a Si(Li) detector from EDAX Inc. The steady state absorption-transmission spectra were obtained with a Perkin-Elmer UV-vis spectrophotometer and PL using an excitation of 266 nm while ultrafast absorption-transmission spectroscopy was carried out using a Ti:Al_2_O_3_ ultrafast amplifier generating 100 *fs* pulses at 800 nm and repetition rate of 1 kHz. Non-linear crystals were used to generate 266/400 nm for the purpose of exciting the Sn-doped In_2_O_3_ NWs whereas part of the fundamental pulse was used to generate a super continuum light for probing different energy states. Finally, Sn-doped In_2_O_3_ NWs were also grown at 500 °C on 10 × 20 mm soda lime glass (SLG) slides which were cleaned as described above followed by the deposition of 1 nm Au after which they were treated under H_2_S at 100, 200, 300, 400, and 500 °C and their resistance measured with a Kiethley 2635 A in accordance with O’Dwyer et al. [18].

## Results and discussion

**Fig. 1:**
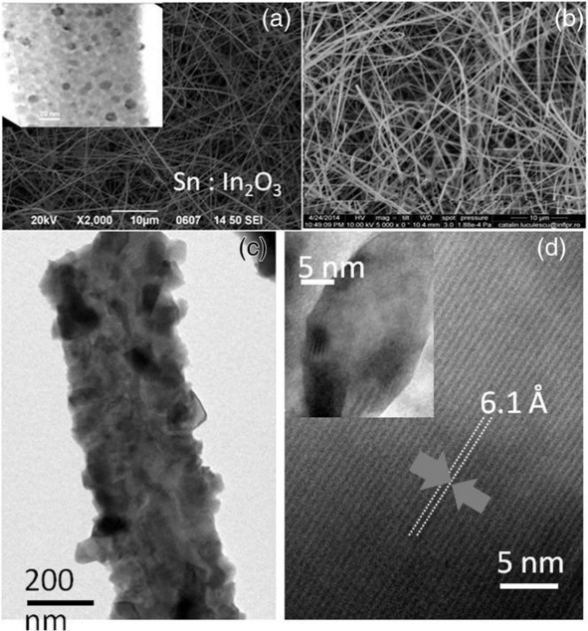
**a** SEM images of Sn:In_2_O_3_ NWs on Si(001). i*nset* shows SnO_2_ nanoparticles on the surface of the nanowires (**b**) SnS_2_:In_2_O_3_ NWs obtained from Sn:In_2_O_3_ NWs under H_2_S at 300 °C (**c**) high magnification TEM image of the Sn:In_2_O_3_ NWs processed under H_2_S at 400 °C (**d**) HRTEM of the crystals shown in **c** giving a lattice spacing of 6.1 Å corresponding to rhombohedral In_2_(SO_4_)_3_

Sn-doped In_2_O_3_ NWs were grown by the VLS mechanism on 1 nm Au/Si(001) at 800 °C using 1–5 % Sn [11]. A typical SEM image of the Sn-doped In_2_O_3_ NWs is shown in Fig. 1a. Their diameters varied between 50 to 100 nm as shown by the inset in Fig. 1a while their lengths reached up to 100 μm. We have shown previously that the Sn-doped In_2_O_3_ NWs have the cubic bixbyite crystal structure of In_2_O_3_ as confirmed by GIXD but also by high resolution transmission electron microscopy (HRTEM) analysis which showed that the lattice spacing is equal to 0.718 nm and corresponds to the d-spacing of the {−1,1,0} crystallographic planes of the cubic bixbyite crystal structure of In_2_O_3_ [11]. However, we also observed the formation of SnO_2_ nanoparticles with a tetragonal rutile crystal structure on the surface of the Sn-doped In_2_O_3_ NWs as shown by the inset of Fig. 1a which is attributed to the limited miscibility and different ionic radii of Sn and In. We do not observe the fluorite structure of In_x_Sn_y_O_3.5_ as shown recently by Meng et al. [19] who observed a flux-induced crystal phase transition in the VLS growth of Sn-doped In_2_O_3_ NWs. The PL of the Sn-doped In_2_O_3_ NWs was found to be broad with a maximum at *λ* = 500 nm or 2.5 eV that shifts to 450 nm or ≈2.8 eV upon reducing the content of Sn to 1 % which also results into an increase in the carrier lifetime as shown previously by time-resolved PL [11]. The PL of the as-grown Sn-doped In_2_O_3_ NWs at 2.8 eV, which is shown in Fig. 3, is not related to band edge emission from In_2_O_3_, which is an n-type semiconductor with a direct energy band gap of 3.5 eV and a lower indirect gap of 2.6 eV. Instead, the PL at 2.8 eV is attributed to radiative recombination related to oxygen vacancies and states residing energetically in the upper half of the energy band gap of In_2_O_3_ as we have shown previously from ultrafast absorption-transmission spectroscopy. Furthermore the n-type Sn-doped In_2_O_3_ NWs had metallic-like conductivities and resistances up to 100 Ω determined from the linear I–V characteristics, shown as an inset in Fig. 3, due to the larger carrier densities of the order of 10^19^ to 10^20^ cm^−3^ [18].

**Fig. 2:**
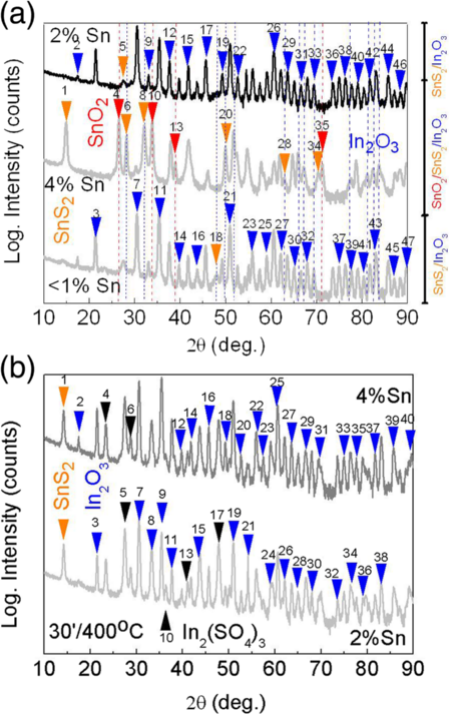
**a** GIXD diffraction pattern of Sn:In_2_O_3_ NWs containing <1 % Sn, 2 % Sn, and 4 % Sn that were exposed to H_2_S at 300 °C for 60 min. The peaks have been labeled with increasing angle in ascending order as follows                                                  and  **b** GIXD diffraction pattern of Sn:In_2_O_3_ NWs containing 2 % Sn and 4 % Sn that were exposed to H_2_S at 400 °C for 30 min                                         The diffracted peaks are labeled by    in ascending order and increasing angle

**Fig. 3:**
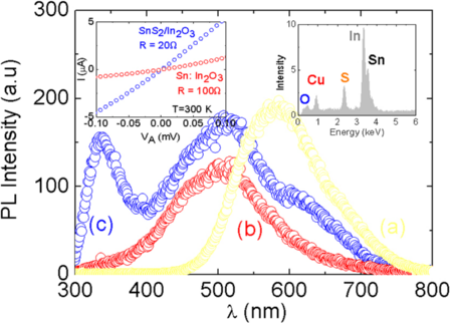
PL spectra of SnO_2_ (**a**), Sn:In_2_O_3_ (**b**), and SnS_2_:In_2_O_3_ (**c**) NWs obtained from Sn:In_2_O_3_ exposed to H_2_S at 300 °C for 60 min, taken at 300 K. *Left inset* shows the I–V characteristic of the Sn:In_2_O_3_ and SnS_2_:In_2_O_3_ NWs; *right inset* shows EDX spectrum of the Sn:In_2_O_3_ NWs processed at 400 °C

We consider next the structural, electrical, and optical properties of the Sn-doped In_2_O_3_ NWs treated under H_2_S between 300 to 600 °C. It is well known that H_2_S undergoes complete decomposition on the surface of oxides even at room temperature and the S atoms bond to the metal cations of the surface. The ionic radii of O^2−^ and S^2−^ are 1.32 and 1.82 Å, respectively, so we expect that S^2−^ will substitute O^2−^ or fill in vacancies. We find that the Sn-doped In_2_O_3_ NWs processed under H_2_S at 300 °C consist mainly of cubic bixbyite In_2_O_3_, tetragonal rutile SnO_2_, and hexagonal SnS_2_ as shown by the GIXD in Fig. 2a where the peaks have been identified according to ICDD 01-071-5323 for SnO_2_, ICDD 00-023-0677 for SnS_2_, and ICDD 04-012-5550 for In_2_O_3_. More specifically, we find that the Sn-doped In_2_O_3_ NWs with 1–2 % Sn are converted into SnS_2_/In_2_O_3_ NWs at 300 °C while we observe SnO_2_, SnS_2_, and the dominant cubic bixbyite In_2_O_3_ after exposing the Sn-doped In_2_O_3_ NWs containing 4 % Sn to H_2_S at 300 °C also shown in Fig. 2a. A typical EDX spectrum of the Sn-doped In_2_O_3_ NWs processed under H_2_S at 400 °C confirming the presence of In, Sn, and S is shown as an inset in Fig. 3.

Similarly we observe the cubic bixbyite In_2_O_3_ but also the emergence of rhombohedral In_2_(SO_4_)_3_, identified using ICDD 00-027-1163, in the GIXD of the Sn-doped In_2_O_3_ NWs containing 2–4 % Sn after post growth processing under H_2_S at 400 °C as shown in Fig. 2b. A high magnification TEM image is shown in Fig. 1c from which one may observe the formation of the In_2_(SO_4_)_3_ crystals on the surface of the Sn-doped In_2_O_3_ NWs with a lattice spacing of 6.1 Å determined from the HRTEM of Fig. 1d and identified using PDF 83–217. The Sn-doped In_2_O_3_ NWs did not remain one dimensional above 400 °C probably due to their rapid reduction by the H_2_ evolving from the decomposition of H_2_S which requires high temperatures in the range 750 to 1250 K. Hence, we consider further the properties of the Sn-doped In_2_O_3_ NWs processed under H_2_S below 500 °C.

**Fig. 4:**
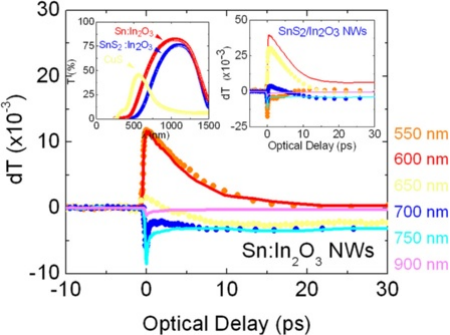
Ultrafast transient spectroscopy of ITO. *Right inset* shows the ultrafast transient of the SnS_2_:In_2_O_3_ NWs obtained from the ITO NWs under H_2_S at 300 °C for 60 min; *left inset* shows the steady state absorption-transmission spectra of the ITO and SnS_2_:In_2_O_3_ NWs

The Sn-doped In_2_O_3_ NWs exposed to H_2_S at 300 °C exhibited PL at *λ* = 340 nm or 3.4 eV as shown in Fig. 3 corresponding to band edge emission from In_2_O_3_. The emergence of band edge emission at ≈3.4 eV is still accompanied by the broader PL around 500 nm or 2.5 eV observed in the as-grown Sn-doped In_2_O_3_ NWs. The emergence of band edge emission is attributed to a suppression of the surface recombination similar to what has been observed in the case of bulk ZnO [7]. Here, it should be noted that SnS_2_ is an indirect band gap semiconductor but exhibits defect-related PL around 2.0–2.5 eV as we have shown recently by post growth processing of SnO_2_ NWs under H_2_S [20]. In addition, note that InS has an indirect energy gap of 1.9 eV, β-In_2_S_3_ is an n-type semiconductor with a direct band gap of 2.1 eV while it has been found that the optical band gap varies from 2.1 eV in pure β-In_2_S_3_ to 2.9 eV in β-In_2_S_3-3x_O_3x_ when it contains 8.5 at.% of oxygen [13]. Consequently the PL the Sn-doped In_2_O_3_ NWs at ≈3.4 eV is related to the cubic bixbyite In_2_O_3_ which is dominant after post growth processing under H_2_S between 300 to 400 °C. For completeness, the steady state transmission through the Sn-doped In_2_O_3_ NWs grown on fused silica at 800 °C before and after post growth processing under H_2_S at 200 °C is shown as an inset in Fig. 4. One may observe a slight reduction in the maximum transmission and a small red shift, but the overall shape has not changed, and the maximum occurs at *λ* ≈ 1000 nm. This red shift is consistent with ultrafast, differential absorption-transmission spectroscopy measurements, shown in Fig. 4. The differential transmission through the Sn-doped In_2_O_3_ NWs grown on fused silica between *λ* = 550 to 600 nm is positive and decays over a few tens of ps, but we observe a suppression of the *λ* = 550 nm trace and increase in differential transmission around *λ* = 650 to 700 nm after post growth processing under H_2_S as shown by the inset in Fig. 4. This is attributed to the formation of SnS_2_ on the surface of the Sn-doped In_2_O_3_ NWs and is responsible for the red shift observed in the steady state transmission spectrum.

In addition to the optical properties, we find that the Sn-doped In_2_O_3_ NWs had resistances of ≈100 Ω, determined from the I–V characteristics shown as an inset in Fig. 3 and maintain their metallic-like conductivity after exposure to H_2_S at 300 °C with resistances of ≈20 Ω.

These findings are important for the realization of p-n junction devices between Sn-doped In_2_O_3_ NWs in contact with p-type chalcogenide semiconductors like Cu_2_S or core-shell Cu_2_S/Sn-doped In_2_O_3_ NWs via the deposition of Cu over Sn-doped In_2_O_3_ NWs followed by processing under H_2_S [17] Similar core-shell Cu_2_S/Sn-doped In_2_O_3_ NWs have been used as sensors or in QDSSCs by Jiang et al. [21] who decorated n-type Sn-doped In_2_O_3_ NWs with p-type Cu_2_S quantum dots (QDs) using solution-processing methods. In such devices the Sn-doped In_2_O_3_ NWs that are not covered with Cu_2_S QDs come into direct contact with polysulfide liquid electrolytes containing S, Na_2_S etc. It is well known that electron-hole recombination at the transparent conducting oxide-liquid electrolyte interface may reduce the overall efficiency. Consequently the deposition of Cu over Sn-doped In_2_O_3_ NWs followed by its conversion into p-type Cu_2_S under H_2_S will result into the formation of a core-shell p-n junction but the surface not covered by Cu will be passivated by sulfur which is compatible with polysulfide electrolytes of QDSSCs. Nevertheless one of the challenges in the fabrication of QDSSCs is to grow the Sn-doped In_2_O_3_ NWs on low-cost transparent substrates such as soda lime glass in order to maintain transparency.

Hence we have grown Sn-doped In_2_O_3_ NWs on 10 × 20 mm glass slides via the VLS mechanism at temperatures below 600 °C in order to prevent bending and melting. We obtained a high-yield, uniform distribution of Sn-doped In_2_O_3_ NWs over the 15 × 20 mm glass slide similar to that shown in Fig. 1a. The Sn-doped In_2_O_3_ NWs had metallic-like conductivities and resistances less than 100 Ω, but the conductivity changed from being metallic to insulator-like after processing under H_2_S above 200 °C which we also observed in the case of ITO films on glass. This deterioration in the electrical resistance and conductivity may be related to Na ion diffusion as it was not observed in the case of the Sn-doped In_2_O_3_ NWs grown on Si. Fortunately the Sn-doped In_2_O_3_ NWs on glass maintain their metallic-like conductivity by processing under H_2_S between 100–200 °C which is sufficient for the conversion of Cu into p-type Cu_2_S and the realization of p-n junctions.

## Conclusions

We have investigated the effect of post growth-processing Sn-doped In_2_O_3_ NWs under H_2_S on their structural, electrical, and optical properties. We observe the existence of hexagonal SnS_2_ and cubic bixbyite In_2_O_3_ which is dominant after exposing the Sn-doped In_2_O_3_ NWs to H_2_S at 300 °C while we also observe the cubic bixbyite In_2_O_3_ and emergence of orthorhombic In_2_(SO_4_)_3_ at 400 °C. All of the Sn-doped In_2_O_3_ NWs maintain their metallic-like conductivity and have resistances between 10 to 100 Ω after processing under H_2_S while we also observed the emergence of PL at 3.4 eV corresponding to band edge emission from In_2_O_3_. Finally, we have grown Sn-doped In_2_O_3_ NWs on glass at 500 °C which may be processed under H_2_S only between 100 to 200 °C which allows the deposition of Cu over the Sn-doped In_2_O_3_ NWs and their subsequent conversion into Cu_2_S/Sn:In_2_O_3_ core-shell p-n junctions for use in QDSSCs.
